# RACE DIFFERENCES IN THE CONSEQUENCES OF ADULT CHILDREN’S PROBLEMS ON MOTHERS’ PSYCHOLOGICAL WELL-BEING

**DOI:** 10.1093/geroni/igz038.2488

**Published:** 2019-11-08

**Authors:** Catherine Stepniak, J Jill Suitor, Megan Gilligan, Karl Pillemer, Marissa Rurka

**Affiliations:** 1 Purdue University, West Lafayette, Indiana, United States; 2 Iowa State, Ames, Iowa, United States; 3 Cornell University, Ithaca, New York, United States; 4 Purdue University, Lafayette, Indiana, United States

## Abstract

Adult children’s problems have been found to be strong predictors of older parents’ psychological well-being, regardless of whether the sources of the problems are psychological or physical health, life circumstances outside of the children’s control, or children’s poor life decisions. Further, this pattern remains regardless of the number or proportion of offspring with problems, or whether children with problems were favored or disfavored by their parents. One important question that has not been addressed is whether the impact of children’s problems differs in Black and White families. Race disparities in health and other life circumstances lead Black adult children to be at greater risk of experiencing problems than are their White counterparts. Thus, Black mothers are at greater risk of having adult children with problems; however, increased exposure does not necessarily lead to a stronger impact of children’s problems on well-being. Alternatively, it can be argued that due to stronger kin networks and higher levels of religiosity, children’s problems may have a weaker impact on Black than White mothers’ well-being. In this paper, we use mixed-methods data collected from 101 Black mothers and 295 White mothers as part of the Within-Family Differences Study to explore differences in the impact of adult children’s problems on mothers’ depressive symptoms. Preliminary analyses of quantitative and qualitative data suggest that mothers’ interpretations of the circumstances surrounding their children’s problems, rather than support or type of problem, play a greater role in the impact of those problems on well-being in Black than White families.

